# The role of inhibition in modeling decision making with spiking neural networks

**DOI:** 10.3389/fninf.2026.1854811

**Published:** 2026-08-12

**Authors:** Bartłomiej Król-Józaga, Peter Duggins, Anna Broniec-Wójcik, Szymon Wichary

**Affiliations:** 1 Department of Biocybernetics and Biomedical Engineering, AGH University of Krakow, Krakow, Poland; 2 Department of Psychological and Brain Sciences, Dartmouth College, Hanover, NH, United States; 3 Institute of Psychology, Jagiellonian University, Krakow, Poland

**Keywords:** decision making, inhibition, neural engineering framework, spiking neural networks, strategy use

## Abstract

Decision making (DM) requires coordination of elementary information processes subserved by a distributed network of brain areas. Computational models help to understand these processes, but most of the existing models focus on simulating only one of the many parallel operations. An existing spiking neural network (SNN) model attempts to simulate DM holistically, however it does not take advantage of the significant role of inhibition at the neural level as a possible mechanism underlying DM. To address this limitation, we propose to examine the impact of neural inhibition on decision strategy selection in value-based DM using the mentioned model. In this study we outline the methodology and perform successful in-silico validation of the inhibition hypothesis with the SNN model of DM. To perform the simulation, we use a well-studied multi-attribute choice task and we validate simulation results against human behavioral data. The inhibition model achieved approximately 17% lower mean prediction error than the no-inhibition model (0.55 vs. 0.67) when evaluated on held-out, compensatory-condition data not used for fitting (Wilcoxon signed-rank test, *p* = 0.009, *r* = 0.55), with no significant difference observed in the condition used for fitting. These findings indicate that the advantage conferred by inhibition is not attributable to model complexity alone, and support neural inhibition as a plausible, biologically grounded mechanism for adaptive, context-sensitive decision strategy selection.

## 1 Introduction

Decision making (DM) requires coordination of multiple neural subprocesses, such as reward processing, evidence accumulation, decision strategy use and option selection (Gold and Shadlen, 2007). One way to understand these intertwined processes is by building artificial neural networks that try to mimic brain functions by implementing particular neural features, such as inhibition, in order to produce human-like choices. The aim of this paper is to investigate pre-decisional information processing and decision strategy use and the role that inhibition at the neural level plays in these processes, by using a spiking neural network (SNN) model.

Normative theories of choice postulate that decision makers should process all available information, integrating attribute weights and values into overall valuations of choice options. In contrast, descriptive theories of choice postulate that in order to simplify choice

problems, decision makers often use heuristics (Gigerenzer and Todd, 2001), which trade off accuracy for speed. Given their simplicity, heuristics were proposed as plausible models of choice under various conditions, such as scarce knowledge, memory limitations, time pressure or stress (Rieskamp and Hoffrage, 2008; Wichary et al., 2016).

Computational models have explored many aspects of DM, including the speed-accuracy tradeoff (Bogacz et al., 2006) and the use of rational strategies vs. heuristics (Bergert et al., 2007; Lee and Cummins, 2004). However, there is still very little understanding of the underlying neural mechanisms of these processes. Inhibitory mechanisms have been incorporated into diffusion-like and accumulator models of DM (López and Pomi, 2024; Zemliak and MacInnes, 2022), yet their implementation in biologically detailed spiking neural network models to account for high-level phenomena such as decision strategy use remains rare. Early models of elementary cognitive processes, e.g., attention (Servan-Schreiber et al., 1990; Usher et al., 1999) have studied this aspect, however this did not translate into the development of computational models of complex DM.

To understand how choice processes are produced by neural activity and map this onto a SNN model, we draw from the growing neuroscience research on DM. In the previous SNN model of complex DM, (Duggins et al., 2020) reviewed lines of evidence suggesting that brain areas such as orbitofrontal cortex (OFC) (Clithero and Rangel, 2014), dorsolateral pre-frontal cortex (dlPFC) (Thura and Cisek, 2014) and basal ganglia (BG) (Bogacz and Gurney, 2007; Frank, 2006) play important roles in DM by binding sensory input with value and relevance signals, as well as accumulating, maintaining and integrating information over time and selecting the best choice option. Areas such as anterior cingulate cortex (ACC), inferior frontal cortex (IFC) and pre-supplementary motor area (preSMA) are also involved, modulating choice processes by shifting decision thresholds as a response to control demands (Aron et al., 2014; Forstmann, 2010).

Inhibitory cortical connections are essential for complex cognition, as they regulate brain activity to maintain the excitation-inhibition balance. Research (Kann et al., 2014) on inhibitory interneurons demonstrates their role in synchronizing neural oscillations that are key to coordinating the networks involved in choice processes. By filtering irrelevant information (Roux and Buzsáki, 2015), inhibition helps refine neural responses, allowing adaptive DM under varying cognitive demands. This mechanism supports the interaction between regions such as OFC, dlPFC and BG, which are central to accumulating, integrating, and selecting the best choices based on relevance and value signals. These mechanisms ensure cognitive flexibility and control, allowing for adaptive DM while avoiding pathological over-excitation, which could impair cognitive and brain functions, as seen in conditions like epilepsy or schizophrenia. The work of Isaacson and Scanziani (Isaacson and Scanziani, 2011) further underscores the role of lateral inhibition in refining neural circuits during complex tasks, helping to fine-tune responses for optimal DM. Research by Aron et al. (2014) has also highlighted the importance of inhibitory connections in the right inferior frontal cortex in maintaining cognitive flexibility and preventing runaway excitation that could lead to impaired DM. In addition, the research on response inhibition (Aron et al., 2014) has highlighted the role of the brain inhibition network, with areas such as IFC and preSMA involved in cognitive control processes that regulate decision making (Venkatraman et al., 2009).

To study the role of inhibition in strategy use in complex DM empirically, we used the sequential probabilistic inference task (Wichary et al., 2017; Cacek et al., 2026). Participants begin performing this task by memorizing the validities (weights) of six attributes. The weights in this task are either Compensatory (C)—they do not differ significantly from one another, encouraging participants to attend to each attribute, or Non-Compensatory (NC), encouraging participants to heuristically discard the low-weight attributes (Martignon and Hoffrage, 1999). During the task, participants simultaneously see the values (0 or 1) of choice options A and B for one attribute at a time. The participants respond by pressing one of three buttons, indicating their selection of A, selection of B, or a request for more information. If they request another attribute, the current display is replaced by another pair of attribute values. This is repeated until the participant makes a choice or until all attributes have been exhausted, at which point a choice is forced. Each participant performs the task 36 times per experimental condition (Compensatory vs. Non-Compensatory). Behavior on each trial is quantified by the number of attributes requested before the final decision and whether the choice corresponded to one of two choice strategies: the normative Weighted Additive rule (WADD) (Payne et al., 1988) or the simple heuristic Take The Best (TTB) (Gigerenzer and Goldstein, 1996). In the WADD strategy, all available attributes are considered and weighted by their importance; the option with the highest weighted sum is chosen. In contrast, the TTB heuristic involves examining attributes sequentially in order of importance and selecting the first option that has a higher value on the most discriminating attribute, ignoring the rest.

In this paper, we aim to model pre-decisional information processing in complex decision-making processes and examine the influence of neural inhibition on this process. Our objectives are twofold. First, we hope to implement the cognitive mechanisms of decision-making models in biologically plausible neural networks, using finely tuned parameters that define individual characteristics of different people instantiated within synaptic connection weights. Successfully achieving this goal, we aim to demonstrate that these cognitive mechanisms can be directly instantiated within neural structures that mimic human brain function. Second, we aim to show that such a customized model can reproduce the impact of neural inhibitory activity on the decision-making process and its outcome at the behavioral level. Based on the extant literature on the role of inhibition in cognition (Aron et al., 2014), we assume that inhibitory neural processes modulate decision processes to facilitate the use of complex, rational choice strategies.

**Figure 1:**
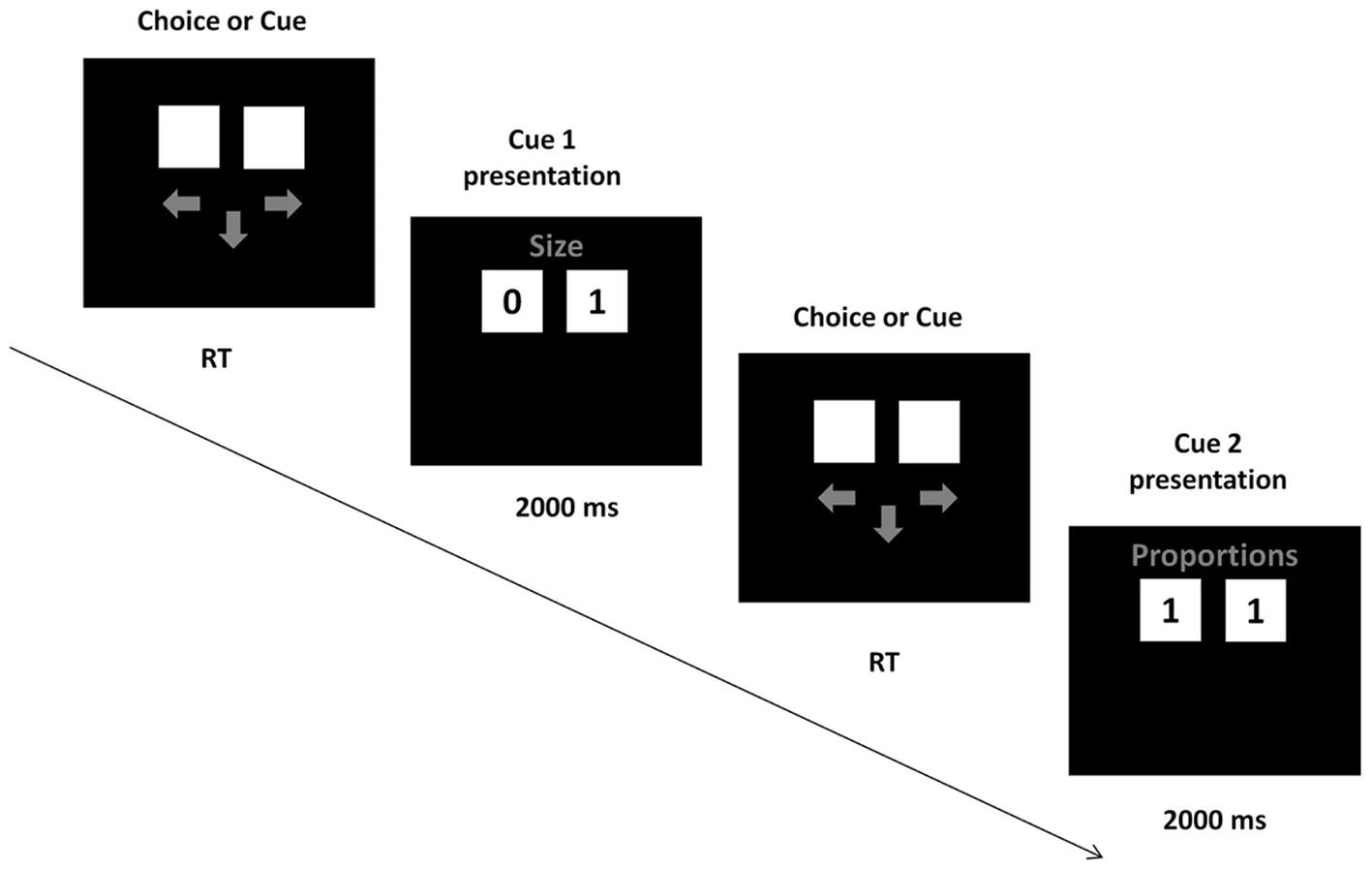
Schematic overview of the decision task. In each trial, participants could either request a cue or make a choice, by pressing the down arrow button (next cue) or left or right arrow buttons (choice). Participants could request up to six cues and a cue was presented for 2,000 ms. On a given trial, a cue either discriminated between the alternatives (one alternative had a “0” value and the other had a “1” value) or did not discriminate (both alternatives had the same cue value) Figure is reproduced (verbatim, 1:1) from: (Wichary et al., 2017). License: CC BY 4.0. Source: Frontiers in Human Neuroscience.

We begin by describing our network model in detail and presenting its basic behavior within the context of the Neural Engineering Framework. We then evaluate the model's performance by comparing its outputs to behavioral data from an experiment on complex decision-making (Figure 1). We validate the model in the context of strategy preference, examining its ability to predict the tendency to select specific decision-making strategies by human decision-makers, namely the rational Weighted Additive rule vs. the simple heuristic Take The Best in a multi-attribute choice task (Wichary et al., 2017). We do it by, first, adapting the basic decision-making model to the Non-Compensatory condition and then validating its behavior in the contrasting Compensatory condition. We then replicate this experiment with the inclusion of the inhibitory connections to the OFC neuron population in our model, where the integration of attribute information with value signals takes place. This, we assume, represents the impact of modulatory inhibitory pathways on the decision process. Based on the research on the role of neuromodulation and inhibition in cognition, we assume that these pathways adaptively respond to various situational demands such as increasing cognitive load. By conducting these modeling exercises, we aim to validate our SNN decision-making model, ensuring its robustness across various conditions. We argue that our model helps to integrate multiple levels of analysis by describing how neural mechanisms in the brain implement the cognitive mechanisms of decision-making, and by confirming the influence of inhibitory neural activity.

## 2 Materials and methods

**Figure 2:**
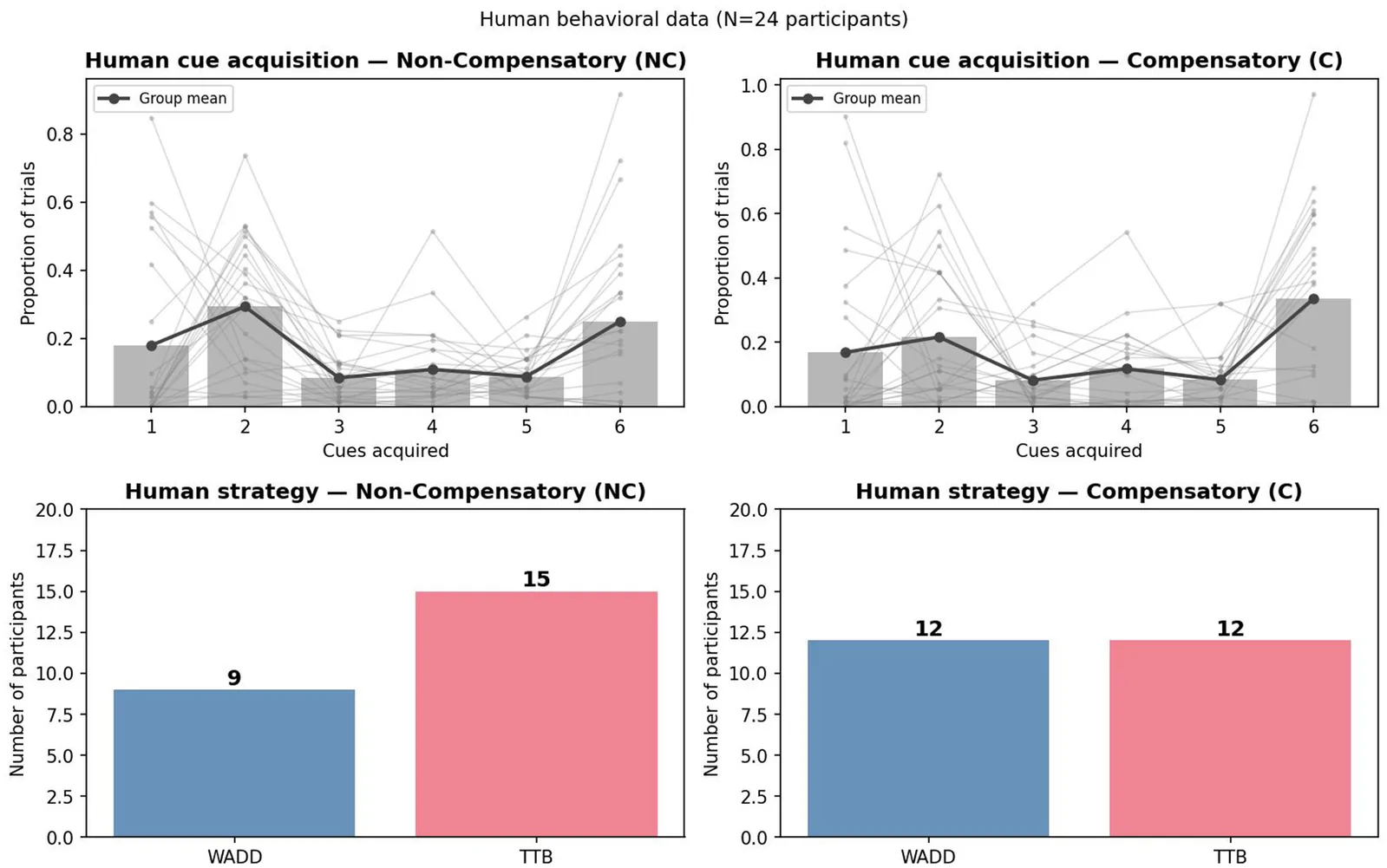
Human behavioral data (*N* = 24 participants). Top row: group-level cue acquisition distributions (bars: group mean; gray lines: individual participants) for the Non-Compensatory (NC, left) and Compensatory (C, right) conditions. Bottom row: number of participants classified as WADD or TTB users under each condition (discriminating-trial classification; see Methods). In NC, most participants use the TTB heuristic (15/24); in C, the distribution is equal (12/24 each), reflecting the greater adaptive value of WADD when cue validities are similar.

Our model was fitted to human behavioral data (*N* = 24, 21 woman, age 21-27) from the sequential probabilistic inference task described in Section 1 (Wichary et al., 2017; Cacek et al., 2026) (see also Figure 1), in which participants acquire between one and six cues before choosing between two options under either a Non-Compensatory (NC) or a Compensatory (C) attribute-weight distribution. Here we summarize the dataset itself, independently of the model. It comprises *N* = 24 participants (IDs 2–14, 19–27, 30–31), each completing 36 tasks per condition (for most participants presented in two repetitions). For every trial we recorded the number of cues acquired (1–6) and the final choice (A or B), from which we derived, per participant and condition, the cue-acquisition distribution and the preferred decision strategy (WADD vs. TTB, classified on discriminating trials only; see Section 3). These behavioral patterns, prior to any model fitting, are summarized in Figure 2.

In the remainder of this section, we present the main principles of the Neural Engineering Framework (NEF) proposed by (Eliasmith 2003)—a method for building large-scale neural models using realistic neurons. Both the decision model proposed in (Duggins et al. 2020) and our current model were developed within this framework.

### 2.1 Neural engineering framework

The realization of the spiking neural decision-making model was done according to NEF. This methodology contains the mathematical basis for the implementation of dynamic biological neural systems functions. At the core of such systems is the activity of neurons, which represents the vector-valued signal *x*(*t*) (such as value, weight, or evidence) that varies over time. The first Principle of NEF-Representation—assumes that each neuron *i* is defined by: the characteristic of the frequency of spikes (preferred direction vector-encoder, *e*_*i*_), unique gain and a bias. The combination of these values indicates the intensity of neuron activity in response to stimulation. The tuning curve describes how much a particular neuron will fire as a function of the input signal (Equation 1):


$$I_{\text{in}}(t)=\alpha_{i}*\left({\text{e}}_{i}\cdot \text{x}\left(t\right)\right)+\beta_{i}$$ (1)

where *I*_in_(*t*) is the current flowing into the neuron, · is the dot product between the encoder and the input vector, α is a gain factor that scales the neuron's sensitivity to the input, and β is a bias current that shifts the neuron's activation threshold. Now, we can estimate the input signal by decoding the pattern of spikes with a temporal filter that accounts for post-synaptic current (PSC) activity. Filtered spike trains are multiplied with decoding weights (determined by minimizing the squared difference between the decoded estimate and the actual input signal) and summed together to estimate the input based on the spikes. The described encoding and decoding process is the Representation in terms of the NEF.

Transformation—the second Principle of the NEF—assumes the implementation of functions transforming the decoded signal. Thanks to this, it is possible to represent the functions of individual parts of the brain in specific processes (e.g., value-weight multiplication). In order to perform transformations, linear decoding is applied to the neural activities (Equation 2):


$$\hat{f}\left(\text{x}\left(t\right)\right)=\sum_{i=0}^{n}a_{i}\left(t\right)\cdot {\text{d}}_{i}^{f},$$ (2)

where *a*_i_ is the spiking activity of neuron *i, n* is the number of neurons, $d_{i}^{f}$ are basis vectors in the output space that represent elementary functions upon which the transformation is performed. The hat notation is used to mean that the computed function is an estimate. The transformation rule is used to distinguish the decoding process defined by the Representations rule. In its understanding, the value of **x** is encoded by a certain population, and the decoder estimates the value of **x**. In terms of transformation, the decoder estimates the value of *f* **(x)**. In other words—we define a decoder which, instead of extracting the signal represented by the population, estimates the transformation of this signal. From the low-level perspective, optimization of decoders implementing a specific transformation is performed by Nengo neural simulator (Bekolay et al., 2014). Finally, for simulation purposes, we determine single weight values between each pre-synaptic neurons *i* and post-synaptic neurons *j*, which combine encoders and decoders (Equation 3):


$$w_{ij}=\alpha_{j}{\text{e}}_{j}\cdot {\text{d}}_{i}^{f}.$$ (3)

**Figure 3:**
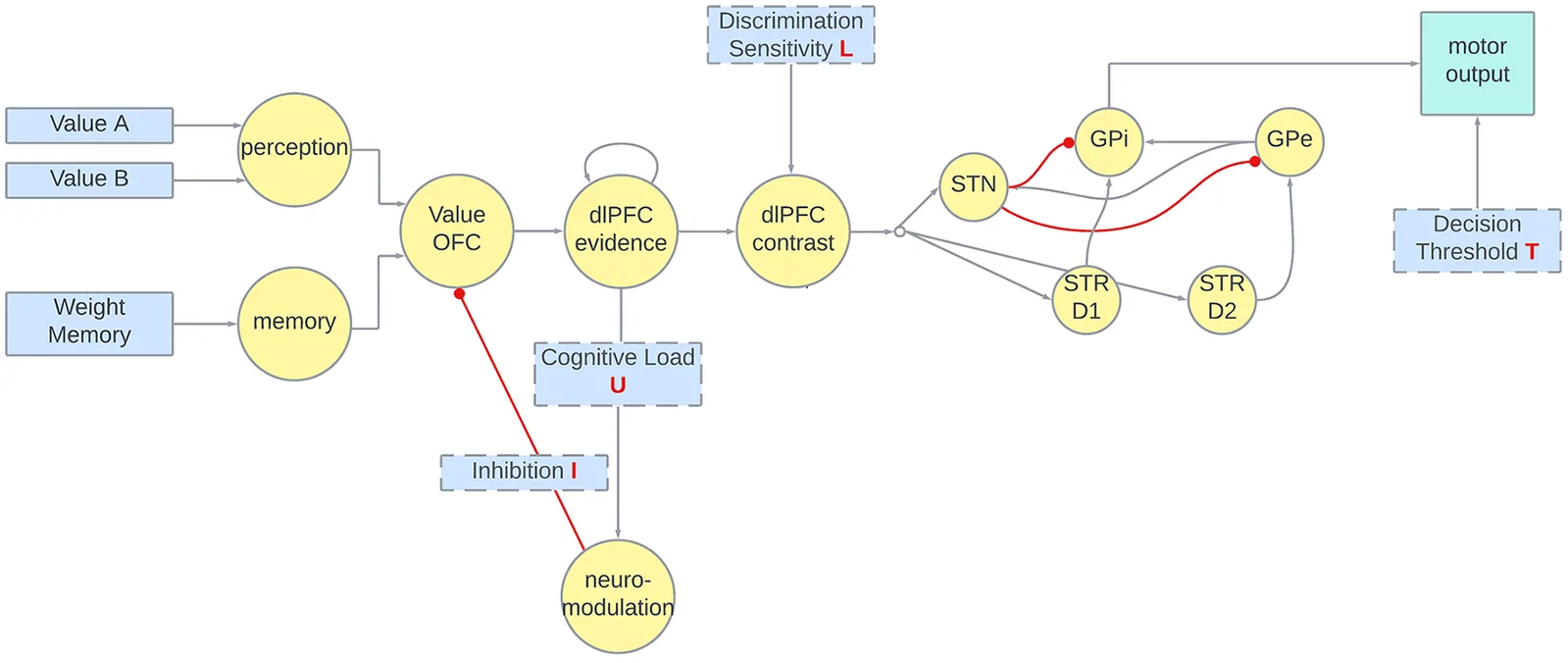
Model schematic. Boxes are inputs and outputs, circles are spiking neuron populations. Red connections are inhibitory. If a box is outlined with a dashed line, it indicates that its value is set within a specified range using Optuna's grid search. See text for details on represented quantities and cognitive operations. OFC, orbitofrontal cortex; dlPFC, dorsolateral pre-frontal cortex; STN, subthalamic nucleus; GPi, globus pallidus pars interna; GPe, globus pallidus pars externa; STR D1, striatum D1 pathway; STR D2, Striatum D2 pathway.

The last element of NEF is Dynamics, which is a response to the treatment of neuronal responses by neuroscientists in terms of stimulus intervals, steady states, or latencies. Any, even the simplest, nervous system is dynamic in its nature, which demands taking time into consideration. At NEF, we define dynamic systems in form ẋ(*t*) = *A***x**(*t*)+*B***u**(*t*) —this equation says that the change in *x*(*t*) (written as ẋ(*t*) is equal to its current value plus the input *u*(*t*) * some number B. Matrices A and B must be modified to account for the effect of the transfer function, which captures the dynamics of biological networks. Nengo performs this optimization for the target dynamics for us. Thanks to this property, we are able to capture mechanisms that use, for example, feedback, such as working memory, choice competition or evidence accumulation (see Figure 3).

In summary, NEF is primarily a framework for dealing with holistic modeling of cognitive processes. It is a convention that does not limit the researcher to isolated mechanisms. It allows to represent the process at low level abstraction (representation), forcing the preservation of the functions performed by the neural connections (transformation), all while maintaining the dynamics of the process (dynamics). In this paper we show that by working in accordance with the three principles of NEF, we can propose an agent that performs behavioral tasks analogically to real participants: input data consistent with those received by the participants of the task; performing measurable functions, comparable with physiological data; returning as output responses that can be mapped to the behavioral decisions of the task participants.

### 2.2 SNN decision making model

Spiking Neural Networks (SNNs) have been applied to various fields, including image recognition, speech recognition, and natural language processing. Unlike traditional artificial neural networks, SNNs are uniquely suited to process temporal information, making them particularly useful for tasks that involve time-varying signals. In our model, the input consists of two vectors representing the values of six cues, for option A and for option B (e.g., successively processed six attributes of two diamonds). Along with their binary values (0 or 1), each cue is associated with a pre-defined weight. These values are processed through the perception and memory components and passed to the population dubbed orbitofrontal cortex (OFC) in our model, where they are combined with the weights stored in memory. The weighted cue values are then forwarded to the population dubbed dorsolateral pre-frontal cortex (“dlPFC evidence”) in our model, where they contribute to the aggregation of evidence over time. This process aligns with previous work on neural evidence accumulation, which suggests that regions like the dlPFC are crucial for integrating information over time during decision-making tasks (Gold and Shadlen, 2007).

In our model, the recursive connections in the “dlPFC evidence” serve as a memory mechanism that integrates and accumulates information across time steps. This aggregation process plays a critical role in decision-making, as previously introduced in models of working memory (Eliasmith, 2013). Further downstream, the model mimics another function of the pre-frontal cortex, namely coding integrated values of choice options and the sensitivity of discrimination (L) between these values. The “dlPFC contrast” population in our model computes the difference between the integrated values of choice options A and B using the formula *A*−*L*·*B* or *B*−*L*·*A*. The purpose of this computation is to enhance the contrast between the option values before passing the signals further, to the Basal Ganglia. This enhancement aims to support the decision-making process by minimizing the influence of signal fluctuations arising from the dynamic properties of neurons and the synaptic filtering mechanisms, while simultaneously providing a participant-specific sense of distinction between the cues.

Finally, the Basal Ganglia (BG) network (Gurney et al., 2001a,b; Stewart et al., 2012), comprising the subnetworks of striatum (STR), globus pallidus internus (GPi), globus pallidus externus (GPe), and subthalamic nucleus (STN), implements a Winner-Take-All (WTA) mechanism. This network architecture is based on well-established models of action selection in the basal ganglia, where competing inputs are resolved through a WTA mechanism (Stewart et al., 2012). Once the preSMA passes the signal to BG, the BG selects the option with the highest accumulated evidence, determining the final decision. The motor output is triggered once the decision threshold (T) is reached, resulting in the model's action.

It is important to note that the BG module used here is the standard nengo.networks.BasalGanglia implementation, which realizes the Gurney et al. (2001a,b) model within the NEF (Stewart et al., 2012). In the NEF, connections between neural populations implement transformations on *decoded* population representations rather than mapping directly onto individual biological synapses. Consequently, the sign of a connection's transform matrix reflects the direction of the mathematical transformation applied to the represented quantity, not the neurotransmitter type of the underlying synapse. The canonical GABAergic projections of GPe and GPi are correctly encoded as subtractive operations on downstream population representations; the glutamatergic STN projections are encoded as additive operations. The resulting WTA dynamics—selective disinhibition of one action channel while suppressing all others—faithfully reproduce the action-selection behavior described in Gurney et al. (2001b) and have been validated against known basal-ganglia electrophysiology (Stewart et al., 2012).

Importantly, in the current version of the model, we introduce neuromodulatory connections to the OFC. In our model, this plays a key role in modulating decision-making by accumulated evidence (information or “cognitive load”). We assume that this modulation stems from the activity of the neuromodulatory systems, particularly the locus coeruleus-norepinephrine system, LC-NE (Aston-Jones and Cohen, 2005) coupled with cortical inhibitory mechanisms (Mather, 2016). The LC-NE is known for its role in regulating attention, arousal and cognitive effort and its involvement in decision-making has been highlighted in various studies (Aston-Jones and Cohen, 2005; Sara and Bouret, 2012). In our model, the “neuromodulatory” neuron population combines the joint LC-NE and inhibitory neurons' activity to impact the activity of the OFC population, where the integration of attribute information with importance signals takes place. The neuromodulatory population receives inputs from the “dlPFC evidence” population, reflecting dynamically accumulating information about the choice options. This is modulated by parameter U in our model—the parameter that reflects individual responsiveness to the accumulated evidence. As this value increases, the neuromodulatory population increasingly inhibits the activity of the OFC, simulating the effect of effort-related arousal on the selectivity of pre-decisional information processing (Mather, 2016). This aligns with empirical evidence suggesting that higher arousal can lead to increased attentional selectivity via inhibition, particularly in tasks requiring complex decisions (Arnsten, 2009; Wichary et al., 2016).

### 2.3 Simulation of inhibition

Each neural ensemble in Nengo is characterized by several factors, including the number of neurons, dimension (which corresponds to the number of inputs and outputs), and the tuning curves that describe the neurons' response to input values. The input space typically ranges between (–1, 1), and neurons within the ensemble aim to represent this space effectively, with their responses shaped by encoders. These encoders determine whether the neuron exhibits excitatory or inhibitory behavior toward specific input dimensions. Inhibitory connections in Nengo are modeled by using the NEF to set the connection weights such that spikes in the pre-synaptic population always induce a negative input current in the post-synaptic cells, effectively reducing the activity of the target neurons. This mechanism provides a flexible way to simulate inhibition, allowing specific populations or pathways to suppress activity in response to particular inputs or conditions. For details on the LIF neuron's activation function, including the relationship between input current and spike rates, see (Eliasmith 2003).

In Nengo, neuron tuning curves are initialized with constant parameters and remain static throughout a simulation, which can present challenges when modeling dynamically changing inhibitory processes. We interpret the increasing activity of the dlPFC population, associated with the continuous aggregation of cue value information over time, as a marker of cognitive load. This cumulative activity is summed up within the “neuromodulation” population and mapped onto the strength of inhibition, transmitted through inhibitory connections to the OFC. By doing so, we progressively suppress the incoming cue-value and weight product pairs with increasing inhibitory strength. In physiological terms, we postulate that this approach mirrors the way neuromodulatory and inhibitory mechanisms co-operate in the brain, where increased neuromodulation coupled with inhibition decreases neural excitability, effectively representing the influence of effort-related arousal on decision-making.

### 2.4 Model parameters

The model is characterized by four participant-specific parameters (T, U, L, I) that were fitted to the behavioral data, and a set of fixed parameters that were held constant across all participants and both model variants. The four parameters are instantiated within the synaptic connection weights of the network and capture individual differences in the decision process. Table 1 summarizes each parameter, its biological meaning, the search range, and the grid-search step size used during fitting. All four parameters are dimensionless. The parameters were optimized using Optuna (see Section 3 for the fitting procedure); in the No-Inhibition model, the inhibition strength I was fixed at 0, disabling the LC → OFC pathway.

**Table 1:** Participant-specific model parameters.

Param.	Biological meaning	Range	Step
*T*	Decision threshold in the basal ganglia and preSMA: the accumulated-evidence level required to trigger a motor response via basal-ganglia disinhibition (Forstmann et al., 2008, 2010).	[−3, 3]	0.01
*U*	Urgency / neuromodulatory scaling onto the accumulator: controls how strongly cognitive load (accumulated evidence) modulates the rate of evidence accumulation (Thura and Cisek, 2017).	[0, 2]	0.01
*L*	Lateral-inhibition / relative-evaluation gain in the dlPFC contrast computation *A*−*L*·*B*: determines how strongly the less-valued option suppresses the more-valued one (Bogacz et al., 2006; Wang, 2008).	[0, 1]	0.01
*I*	Inhibition strength from the neuromodulatory population onto OFC neurons (Inhibition model only; fixed at 0 in the No-Inhibition model): the magnitude of direct neural inhibition of OFC cue-value representations (Wang, 2008; Isaacson and Scanziani, 2011).	[0, 5]	0.01

All parameters are dimensionless and were fitted to each participant's behavioral data via Optuna grid search over the indicated range and step.

In the No-Inhibition model, I was fixed at 0.

The remaining parameters were fixed across all participants and conditions. The fixed model parameters were *R* = 0.1, *S* = 0.0, and *E* = 0.0. The network architecture parameters were likewise held constant, including the number of neurons per ensemble (*n*_neurons_ = 300) and the simulation time step (*dt* = 0.001 s).

## 3 Results

### 3.1 Model fitting and validation

Validation of the model's ability to simulate human decision-making began with the fitting of agent-specific parameters. To account for the stochasticity of the optimization process and spiking network initialization, the entire fitting procedure was repeated with three independent random seeds (Optuna TPESampler seeds 0, 1, 2) for each participant and both model variants. All results are reported as mean ± standard deviation (SD) across seeds. Each run involved 100 optimization iterations, utilizing one key metric: loss. The loss was computed as the normalized chi-squared dissimilarity between the cue acquisition distributions of the human agents and the model in the NC condition (Equation 4):


$$\begin{array}{l} L=\overset{6}{\underset{k=1}{\sum }}\frac{{\left(p_{\text{sim}}\left(k\right)-p_{\text{emp}}\left(k\right)\right)}^{2}}{p_{\text{sim}}\left(k\right)+p_{\text{emp}}\left(k\right)} \end{array}$$ (4)

where *p*(*k*) is the proportion of trials in which *k* cues were acquired.

**Figure 4:**
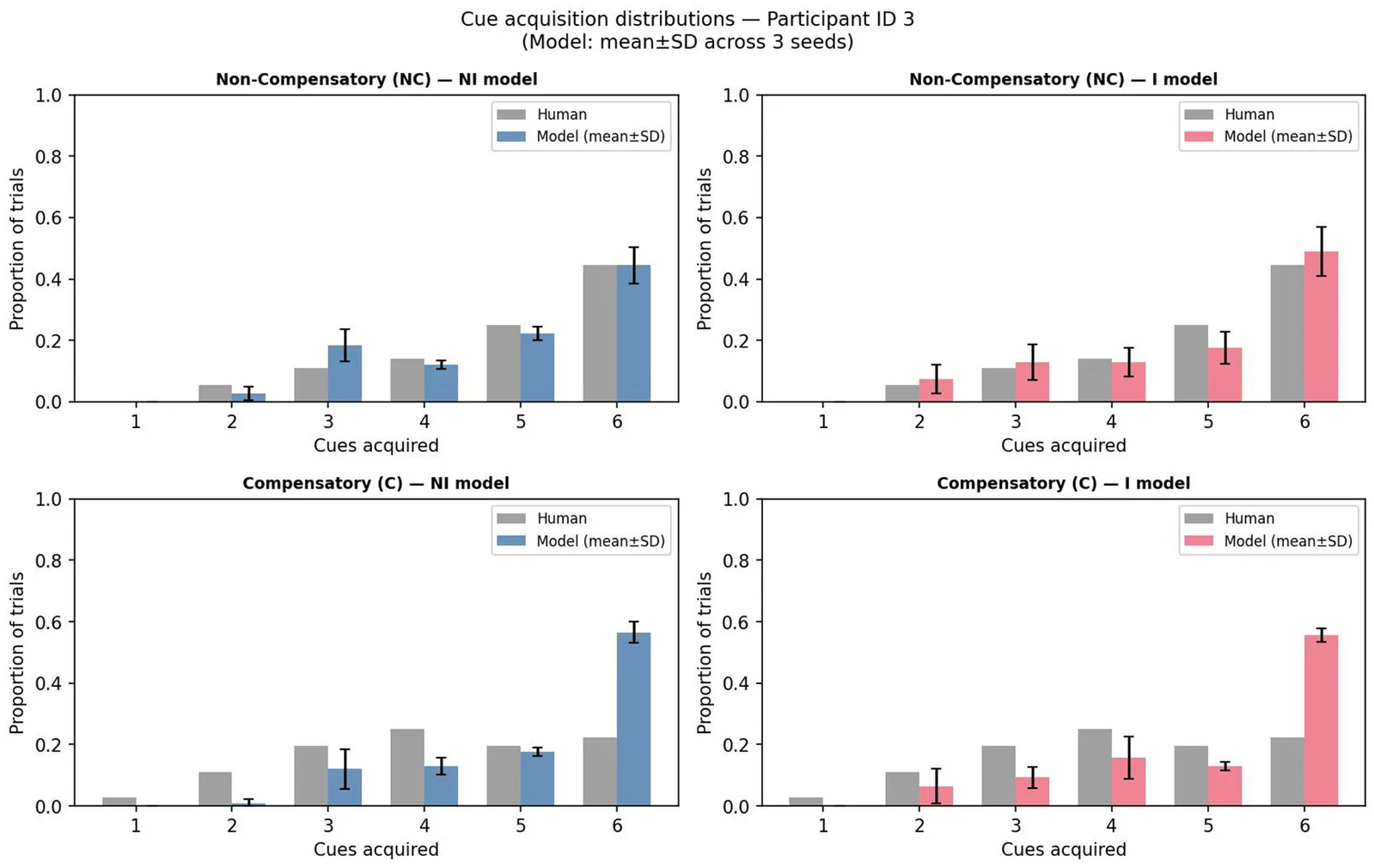
Cue acquisition distributions for illustrative participant ID 3. Each panel compares the human distribution (blue) with the model distribution (mean ± SD across three seeds, red). Rows correspond to conditions (NC, non-compensatory; C, compensatory); columns to model variants (NI, no-inhibition; I, inhibition). Error bars show SD across seeds. Individual-participant results for all 24 participants are provided in the Supplementary material.

Cue acquisition distribution refers to the proportion of trials in which agents, either human or simulated, sampled a given number of cues before making a decision. For example, as shown in Figure 4, these distributions illustrate how frequently agents stopped after sampling N-cues (where N represents the number of cues sampled) under different conditions. By comparing these distributions across conditions, the model's ability to replicate human decision-making patterns could be quantitatively assessed. A modern hyperparameter optimization framework, Optuna (Akiba et al., 2019), was employed for this purpose. Optuna works by efficiently sampling the search space, guiding the optimization toward minimizing the objective function, in this case, the loss.

**Figure 5:**
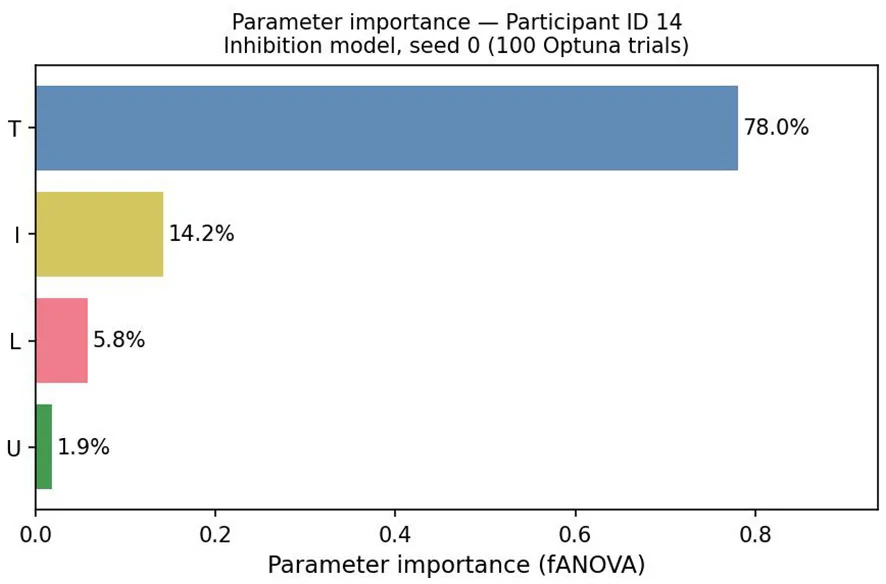
fANOVA parameter importance for illustrative participant ID 14, Inhibition model, seed 0 (100 Optuna trials). Importance values are normalized to the four free parameters (T, L, U, I); fixed parameters (R, S, E) are excluded. T (decision threshold) accounts for 78.0% of the variance in loss, followed by I (inhibition strength, 14.2%), L (5.8%), and U (1.9%).

**Figure 6:**
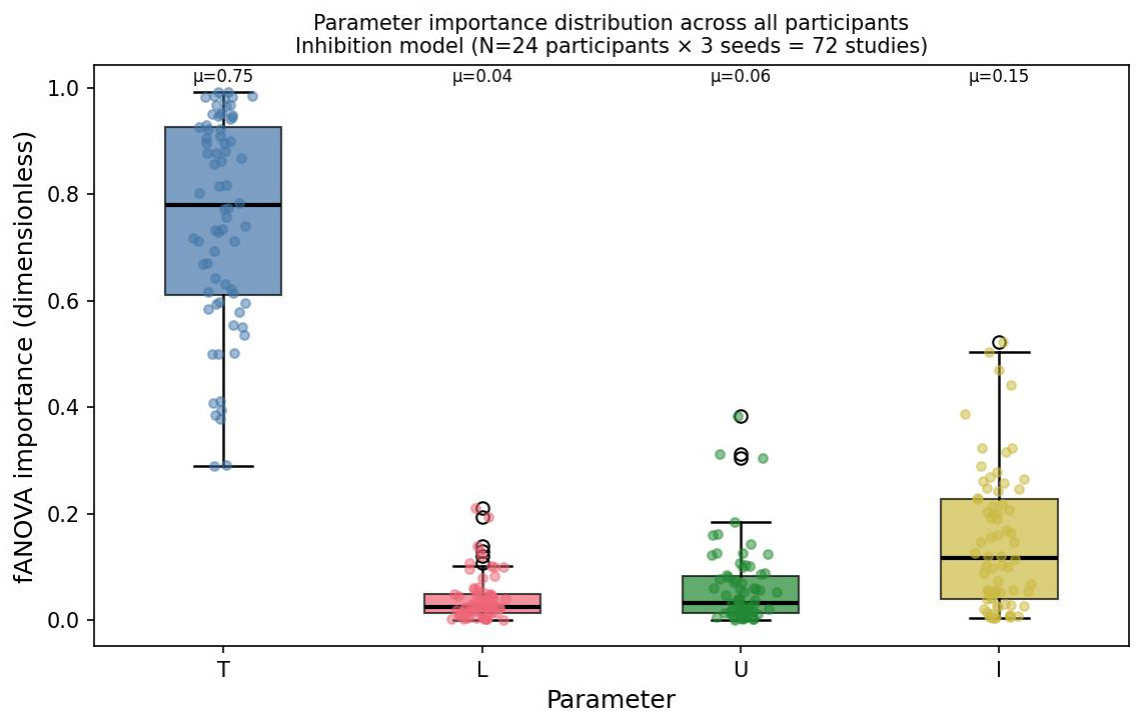
Distribution of fANOVA parameter importance across all 72 optimisation runs (24 participants × 3 seeds) for the Inhibition model. Box plots show median and IQR; individual points are jittered. Group means: T = 0.75 ± 0.20, I = 0.15 ± 0.13, U = 0.06 ± 0.08, L = 0.04 ± 0.05. The decision threshold T is consistently dominant. The inhibition strength I is the second-ranked parameter on average, but shows high inter-individual variability (CV = 87%), indicating it is influential for some participants and negligible for others.

**Figure 7:**
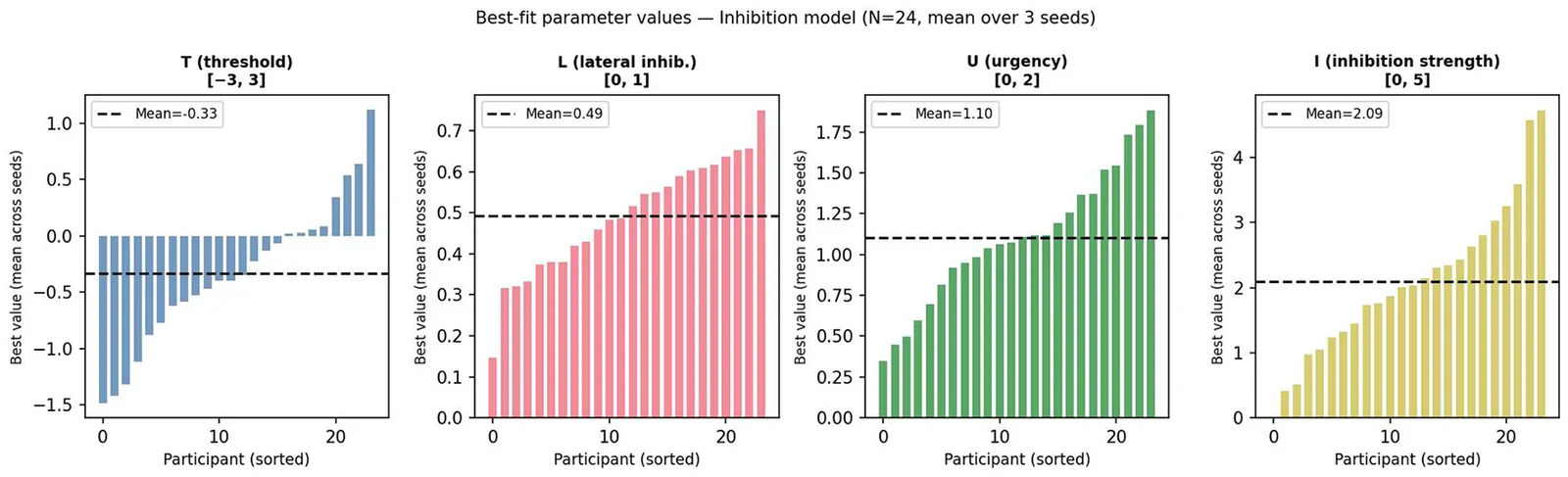
Best-fit values of all four free parameters (T, L, U, I) for the Inhibition model across all 24 participants (mean across three optimisation seeds, sorted by value). Dashed lines indicate group means: T = −−0.33, L = 0.49, U = 1.10, I = 2.09. The inhibition parameter I was greater than zero in 92% of individual seed fits, confirming that the inhibitory mechanism was consistently utilized across the dataset.

In addition to minimizing the loss, the importance of the parameters was monitored using Optuna's built-in fANOVA parameter importance analysis (Hutter et al., 2014). For illustrative purposes, Figure 5 shows the parameter importance for participant ID 14 (one representative seed): the decision threshold T was the most influential (78.0%), followed by the inhibition strength I (14.2%), with L (5.8%) and U (1.9%) contributing minimally. Figure 6 shows the distribution of fANOVA importances across all 72 optimisation runs (24 participants × 3 seeds). T was consistently the dominant parameter (mean = 0.75 ± 0.20). I ranked second on average (mean = 0.15 ± 0.13), but with high inter-individual variability (CV = 87%): for many participants I contributed near zero, while for others it was the decisive second factor. This suggests that the inhibitory mechanism is relevant for a subset of participants rather than uniformly across the group. L and U contributed minimally on average (means 0.04 and 0.06 respectively). Figure 7 shows the distribution of best-fit values of all four parameters across participants (mean across three seeds). Parameter I had a group mean of 2.09 ± 1.17 and was greater than zero in 66 out of 72 individual seed fits (92%), indicating that the inclusion of inhibition was relevant and consistently utilized across the dataset. The threshold T varied widely (mean −0.33 ± 0.64), while L (mean 0.49 ± 0.14) and U (mean 1.10 ± 0.41) showed moderate inter-individual variability.

Fitting was carried out for both the standard decision model and the inhibition model, which accounts for the effect of neuromodulation activity on the orbitofrontal cortex (OFC).

**Figure 8:**
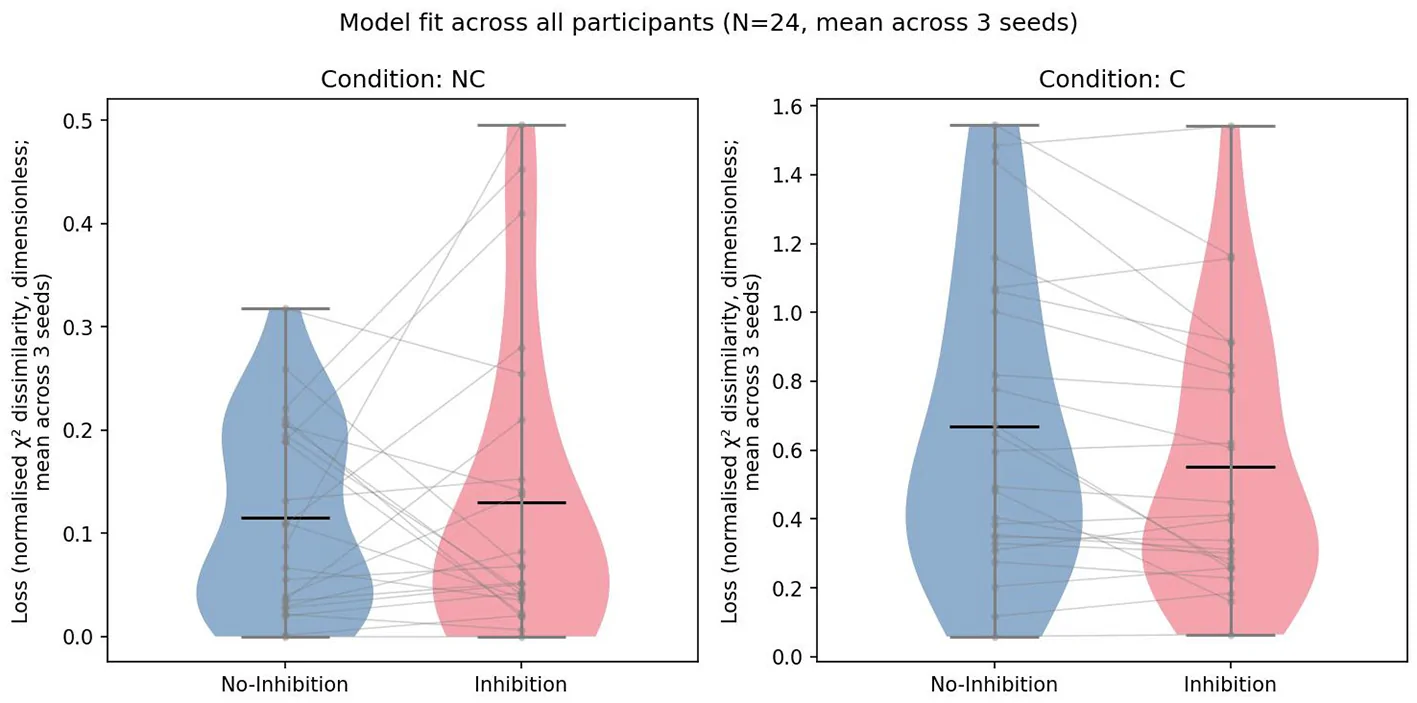
Group-level comparison of model fit across all 24 participants. Violin plots show the distribution of mean loss (averaged across three seeds) for the No-Inhibition (NI) and Inhibition (I) models separately for the NC (training) and C (validation) conditions. Gray lines connect each participant's NI and I mean loss. The Inhibition model does not differ significantly on the training condition (NC, paired *t*-test: *p* = 0.62) but achieves significantly lower loss on the out-of-distribution validation condition (C, Wilcoxon: *p* = 0.009, *r* = 0.55).

As shown in Figure 4, both model variants replicated cue acquisition distributions for participant ID 3 (per-participant results for all 24 participants are reported in Supplementary Table S1, and summarized at the group level in Figure 8). The best-fit parameters from the NC condition were subsequently applied to the C condition (new cue weights) as an out-of-distribution validation. Per-participant loss is reported in Supplementary Table S1 as mean ± SD across three seeds. We adopted this train-on-NC / test-on-C design deliberately, for three reasons. First, it provides the most direct test of our substantive hypothesis: because the C condition uses a more homogeneous set of cue validities that makes rational, attribute-weighting strategies more adaptive while imposing greater cognitive load, transferring the frozen NC parameters to C asks specifically whether the load-dependent LC → OFC inhibitory mechanism–calibrated on the lower-load condition–predicts behavior in the higher-load one. Second, it acts as a built-in control for model complexity: a model that merely exploited its additional free parameter to overfit the NC training data should generalize *worse*, not better, to the unseen C condition. Third, it mirrors the psychological question of whether a decision maker calibrated in one task environment transfers to a structurally different one.

On the NC (training) condition, the two models performed comparably: the No-Inhibition model achieved lower mean loss in 13 out of 24 participants and the Inhibition model in 10 out of 24 [mean loss: NI = 0.115 ± 0.092, I = 0.130 ± 0.143; paired *t*-test: *t*(23) = −0.50, *p* = 0.62, Cohen's *d* = 0.10, 95% CI on mean difference (−0.041, +0.074)]. The difference in fit on the training condition is therefore not statistically significant.

In the C (validation) condition, the Inhibition model generalized significantly better: it achieved lower mean loss in 16 out of 24 participants [mean loss: NI = 0.669 ± 0.431, I = 0.553 ± 0.375; Wilcoxon signed-rank test, one-sided *H*_1_: NI > I: *W* = 232, *p* = 0.009, rank-biserial *r* = 0.55, 95% CI on mean difference (−0.190, −0.049)]. This result supports the hypothesis that the inhibitory LC → OFC mechanism specifically improves the model's ability to generalize to the more cognitively demanding, emotion-laden condition.

**Figure 9:**
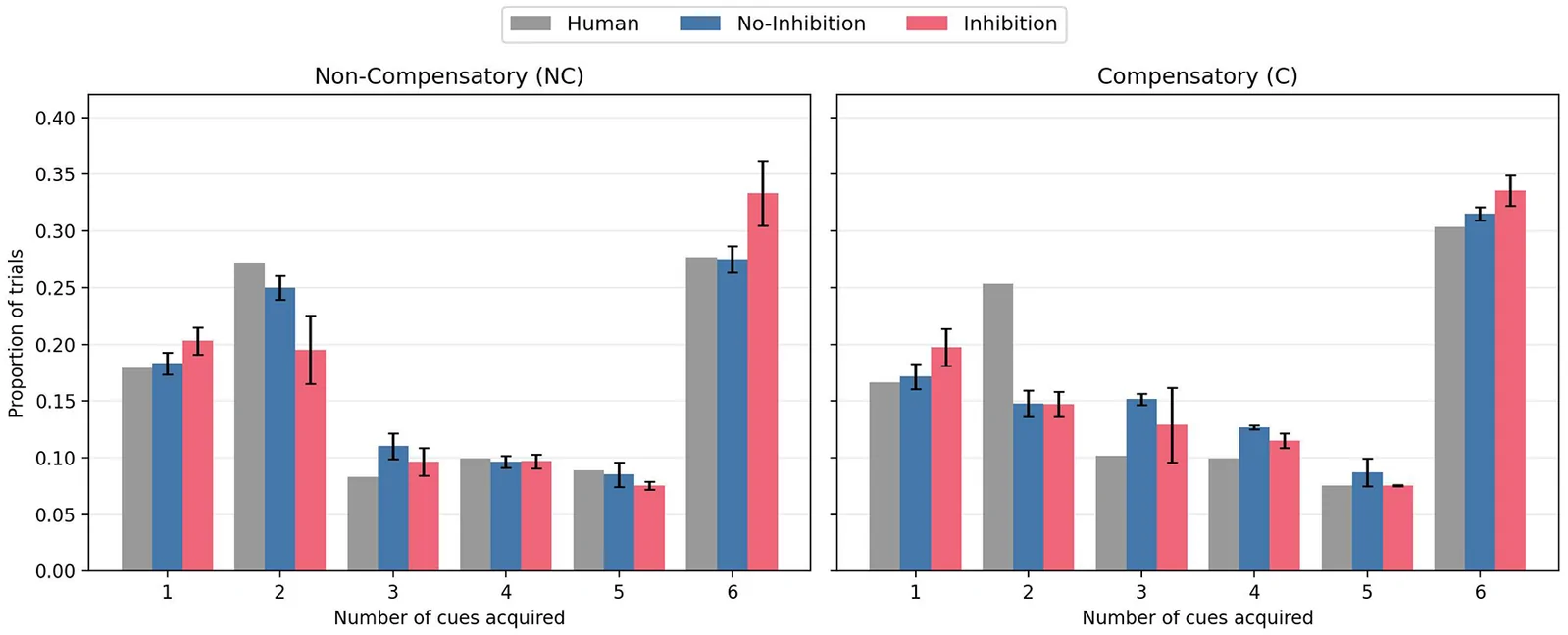
Group-level cue-acquisition distributions: human data (gray) vs. the No-Inhibition (blue) and Inhibition (red) models, for the Non-Compensatory (NC, left) and Compensatory (C, right) conditions. Bars show the proportion of trials on which 1–6 cues were acquired before a decision, averaged across all 24 participants; model bars show the mean across three optimisation seeds and error bars the SD across seeds. Human proportions are the group mean of per-participant proportions. Both model variants reproduce the bimodal structure of the human data, with modes at 1–2 and at 6 cues.

To verify that the models reproduce the characteristic *shape* of the human cue-acquisition distributions—and not merely a low aggregate loss—we compared the group-level distributions directly (Figure 9). Both model variants recover the bimodal structure of the human data, with modes at few (1–2) and at all six cues. In the NC condition the No-Inhibition model closely tracks the human modes at two cues (0.25 vs. 0.27) and at six cues (0.28 vs. 0.28), while the Inhibition model slightly over-represents the six-cue mode (0.33). In the C condition both variants capture the dominant six-cue mode but under-represent the two-cue peak (≈0.15 vs. 0.25 in humans), redistributing that probability mass toward three–four cues. Thus the low aggregate loss reflects a genuine match to the distributional structure—including the diagnostic bimodality of cue sampling—rather than an averaging artifact.

### 3.2 Strategy reproduction

To assess the model's ability to mimic human strategies, we examined its performance with respect to the Take The Best (TTB) and Weighted Additive (WADD) strategies. The comparison in Supplementary Table S1 highlights how models with and without inhibition perform under different conditions (C and NC). The key metric here is the “Model Strategy Preference” column, which shows the preferred strategy for each model, and allows us to see where inhibition led to a better match with human strategies.

The **preferred strategy** is defined as the strategy that was used in the majority of decision tasks across a total of 36 tasks performed by the participants/model. A threshold of 50% or more was used to determine this preference-meaning that if a strategy was employed in at least 50% of the task trials, it was classified as the preferred strategy for that participant or model.

Strategy classification was performed using discriminating trials only—trials on which WADD and TTB predict different choices (47% of NC trials, 36% of C trials)—assigning each participant-model combination the strategy that matched the model's choices more often on those trials. Strategy labels were determined as the majority vote across three seeds.

In the NC condition, both models were pre-dominantly classified as WADD users (NI: 22/24, I: 22/24); a chi-square test found no significant difference between models [χ^2^(1) = 0.00, *p* = 1.00]. Human-strategy agreement was 29% (7/24) for the No-Inhibition model and 38% (9/24) for the Inhibition model; this difference was also not significant [χ^2^(1) = 0.09, *p* = 0.76]. In the C condition, both models showed more balanced strategy use (NI: 11 WADD/13 TTB; I: 12 WADD/12 TTB), more closely matching the human distribution (12 WADD/12 TTB); again no significant difference between models [χ^2^(1) = 0.00, *p* = 1.00]. Human-strategy agreement was 62% (15/24) for NI and 58% (14/24) for I [χ^2^(1) = 0.00, *p* = 1.00].

**Figure 10:**
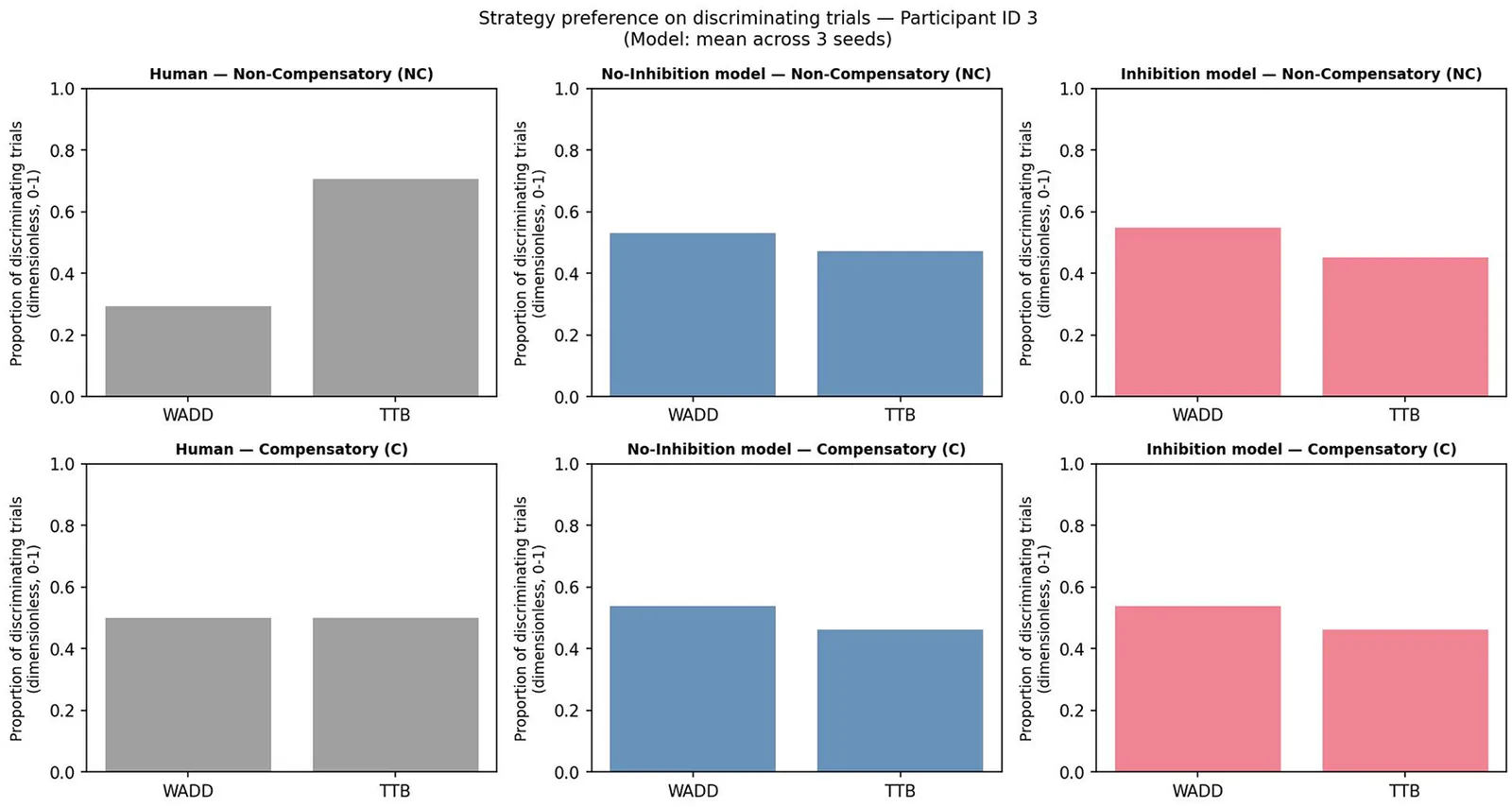
Strategy preference on discriminating trials (trials where WADD and TTB predict different choices) for illustrative participant ID 3. Columns show human data, No-Inhibition model, and Inhibition model; rows show conditions (NC, C). Bar heights represent the proportion of discriminating trials on which the participant/model followed each strategy. Model values are averages across three seeds. Both model variants show similar strategy distributions; the Inhibition model's advantage lies in quantitatively better cue-distribution fit in the C condition (see Figure 8), not a categorical strategy shift.

Figure 10 illustrates an example strategy distribution for participant ID 3 under both model variants and conditions. The group-level strategy results indicate that both model variants predict similar strategy distributions; the advantage of the Inhibition model over the No-Inhibition model lies in its quantitatively better fit to cue acquisition distributions in the C condition (lower loss, *p* = 0.009), rather than in categorically different strategy preferences.

## 4 Discussion

Brain mechanisms of DM are still far from understood. One way of elucidating them is by developing biologically realistic models that can mimic the neural substrates of choice processes. We attempted to do this with an SNN model of inferential DM where humans usually use simple choice heuristics alongside more complex, rational rules.

The results of our study support the hypothesis that a DM model enhanced with a neural inhibition mechanism improves the ability to simulate human behavior in value-based DM, specifically in terms of out-of-distribution generalization. Previous research has shown that brain neuromodulatory activity coupled with inhibition plays a crucial role in mediating the effects of effort and arousal on cognitive flexibility, particularly in tasks requiring complex cognition (Aston-Jones and Cohen, 2005). Our simulations showed that the inhibitory mechanism did not significantly affect model fit on the training condition (NC; *p* = 0.62), but significantly improved generalization to the emotionally demanding validation condition (C; Wilcoxon *p* = 0.009, *r* = 0.55). This pattern is consistent with the view that the LC → OFC inhibitory pathway is specifically recruited under conditions of high cognitive load and arousal, rather than modulating routine decision processes.

### 4.1 Cue distribution and strategy comparison

As indicated by the results concerning cue distribution fit (Supplementary Table S1), the Inhibition model more accurately reflected the distributions of acquired cues in the Compensatory (C) condition—the condition where cue validities are similar, making complex decision rules more adaptive but also imposing greater cognitive load on the decision maker. The difference in the NC training condition was not statistically significant. This dissociation is consistent with findings that the involvement of neuromodulatory systems becomes more critical during tasks with higher cognitive demands (Sara and Bouret, 2012), and specifically supports the role of LC-driven inhibition of OFC as a load-sensitive mechanism.

Regarding strategy reproduction, both models produced pre-dominantly WADD-consistent choices in the NC condition (22/24 participants each), and similar TTB/WADD distributions in the C condition (NI: 11/13; I: 12/12 WADD/TTB). No statistically significant difference between models was found in either condition. This suggests that the quantitative improvement in cue distribution fit in C is not driven by a categorical shift in strategy, but rather by a more accurate replication of the continuous cue acquisition pattern. This finding is consistent with studies indicating that neuromodulation coupled with inhibition (Mather, 2016) shapes selectivity of information processing, and may be proposed as a viable mechanism explaining graded reductions in cue sampling under highly arousing conditions (Wichary et al., 2016).

### 4.2 Model fitting and parameter significance

The fitting process highlighted the importance of specific parameters in both models. In the standard decision model, the threshold (T), which determines the decision point based on attribute differences, was the dominant factor, consistent with traditional decision theories that emphasize the role of thresholds in cue-based decision-making (Busemeyer and Townsend, 1993). In the inhibition-enhanced model, the inhibition strength (I) ranked second on average (mean importance 0.15 ± 0.13), but with high inter-individual variability (CV = 87%): for some participants I was the decisive second factor, while for others it contributed near zero. This suggests that the LC → OFC inhibitory mechanism is relevant for a subset of participants rather than uniformly across the group, and points to meaningful individual differences in the degree to which arousal-based inhibition shapes decision behavior. Where I does contribute, it aligns with prior findings on the interaction between neuromodulation, inhibition and executive functions (Arnsten, 2009; Mather, 2016).

Several alternative explanations for the Inhibition model's advantage warrant consideration. The most immediate is model complexity: the Inhibition model has one additional free parameter (the inhibition strength *I*; the No-Inhibition model is the special case *I* = 0), and a more flexible model can in principle achieve a lower loss for purely statistical reasons. Three features of our results argue against this account. First, the additional parameter did *not* yield a significant advantage on the condition the models were optimized on (NC training condition, *p* = 0.62) - precisely where greater flexibility should help most. Second, the advantage emerged on the held-out Compensatory condition, to which the NC-fitted parameters were transferred without refitting; because added flexibility typically increases overfitting and therefore *harms* out-of-distribution generalization, the complexity account predicts the opposite of the observed pattern. Third, the contribution of *I* was highly heterogeneous across participants (fANOVA importance CV = 87%), being decisive for some and negligible for others, rather than producing the uniform improvement expected from a generic flexibility effect. We nonetheless cannot fully exclude that *I* partially absorbs variance attributable to mechanisms not modeled explicitly here (for instance dynamically changing decision thresholds); formal complexity-penalizing comparisons (e.g., AIC/BIC) or nested cross-validation would further quantify this, although the train-on-NC / test-on-C design already provides a complexity-controlled test of generalization. Finally, the effect could in principle be specific to our cue-acquisition–based loss; however, the convergent strategy-level analysis—no categorical strategy shift, yet a better continuous cue-distribution fit—points to a graded modulation of information sampling consistent with the proposed LC → OFC mechanism rather than an artifact of the fitting metric.

A related question is whether the Inhibition model's advantage in the Compensatory condition reflects genuine generalization across task structures or simply a better fit to that condition. Our design allows us to address this directly. Neither model was refitted in the C condition; instead, both used parameters estimated from NC data. As a result, performance in C reflects out-of-sample transfer rather than within-condition fit. Importantly, this transfer constitutes a more demanding test than conventional split-sample cross-validation because the held-out C trials differ from the training NC trials not only in sampling variability but also in the underlying cue-validity structure. The inhibition parameter did not improve fit in the condition on which it was estimated (*p* = 0.62), yet it produced a better fit when applied to the structurally different C condition. This pattern is difficult to reconcile with an overfitting account, which would predict gains on the training condition but limited benefits when transferring to a new task structure. Consistent with this interpretation, the group-level cue-acquisition distributions (Figure 9) indicate that both model variants capture the characteristic distributional patterns observed within each condition.

### 4.3 Implications, limitations and future work

The results of this study provide evidence for the role of arousal-based neuromodulation coupled with inhibitory mechanisms, in influencing decision-making processes. Incorporating this mechanism into computational models could significantly enhance their ability to replicate complex human behavior, particularly in situations where decisions are driven by more deliberative, attribute-weighting strategies. Similar findings have been reported in studies exploring neuromodulatory influences on cognitive control and decision-making (Doya, 2008; Wang, 2008).

Our modeling exercise has limitations. As any model, ours is of course a crude approximation of the true mechanisms underlying human behavior. For example, in this version of the model, we did not address the question of dynamically changing decision thresholds, which has been discussed in several modeling contexts (Penconek, 2022; Hanks et al., 2014). Given the importance of the threshold parameter in fitting the model to the behavioral data, better modeling of the decision threshold mechanism and integrating it with the rest of the model is desirable, because it could link the model to the empirical studies on brain areas (e.g., preSMA) found to modulate decision thresholds.

**Figure 11:**
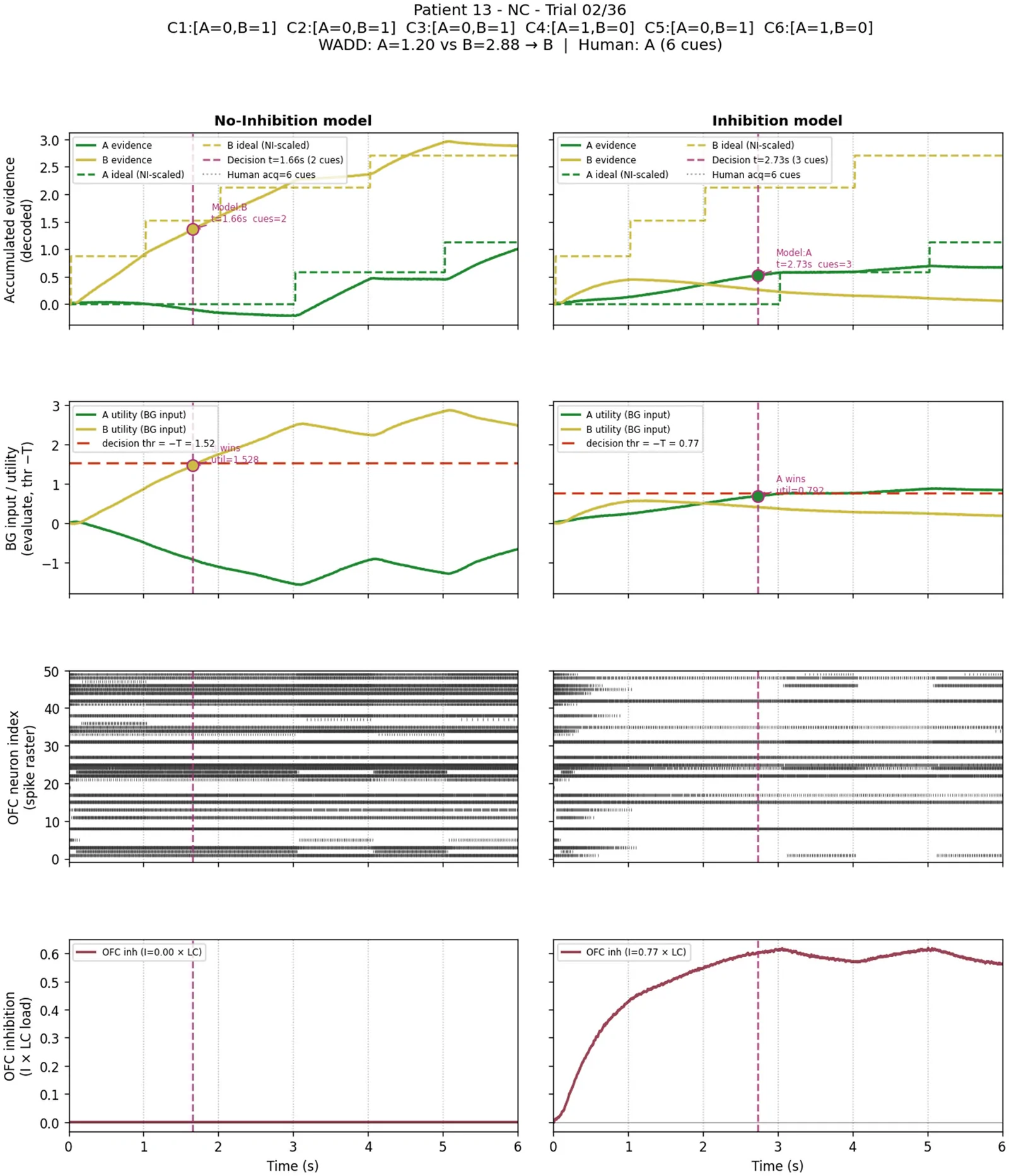
Decoded neural trajectories for participant ID 13, NC condition, trial 2, contrasting the No-Inhibition (NI, left) and Inhibition (I, right) models. Cue pattern: cues 1, 2, 3, 5 favor B; cues 4, 6 favor A (*w* = [0.93, 0.69, 0.64, 0.62, 0.62, 0.58]); WADD favors B (2.88>*A* = 1.20), yet the participant chose A after all six cues. **Row 1:** dlPFC accumulated evidence for A (green) and B (yellow) with ideal weighted-cumulative references (dashed step lines); pink dashed lines mark cue onsets. **Row 2:** BG utility signals and decision threshold −*T* (red dashed); circle = natural threshold crossing; red vertical = forced decision at trial end. **Row 3:** OFC spike raster (50 neurons). **Row 4:** OFC mean output (cognitive-load proxy for LC). In the NI model (*T* = −−1.52, *L* = 0.65), strong lateral inhibition amplifies B's early advantage, nearly suppressing A accumulation throughout. In the I model (*T* = −−0.77, *L* = 0.09, *I* = 0.77), the rising LC signal progressively suppresses OFC activity, attenuating successive cue inputs and permitting A evidence to persist—consistent with the participant's counter-WADD choice. These dynamics illustrate how the LC → OFC pathway gates evidence updates in a load-dependent manner, a mechanism not naturally captured by standard drift-diffusion models without additional neurally grounded structure.

A further architectural limitation concerns the LC cognitive-load signal (Figure 11). In the current implementation, cognitive load is computed as a function of the *accumulated* evidence in dlPFC, which saturates rapidly within the first cue interval due to the strong recurrent feedback in the NEF integrator. A biologically more faithful implementation would derive LC input from the moment-by-moment perceptual processing of individual cues (the OFC population), producing phasic bursts at each cue onset and a gradually increasing tonic baseline across the trial. This distinction matters for interpreting Figure 11: the near-immediate OFC suppression visible in the Inhibition model reflects rapid evidence saturation rather than the trial-by-trial build-up of cognitive load that the LC-NE hypothesis implies. Future work should implement a slow-integrating cue-counter signal for LC and validate the resulting neural trajectories against empirical pupillometry or fMRI data.

Future work could expand on these findings by exploring additional neural mechanisms that influence decision-making, such as the role of other neuromodulators, e.g., dopamine (Frank, 2006). Additionally, further research is needed to refine the inhibition model, particularly with regard to how the strength of neuromodulatory activity varies across different types of decisions and contexts, potentially involving dynamic modulation of arousal in real-time decision-making (Aston-Jones and Cohen, 2005).

It is worth distinguishing which aspects of our model are essential to the scientific claim and which are implementation choices. The essential ingredient is a mechanism that explicitly represents pre-decisional cue/value information (an OFC-like population) and allows a separate neuromodulatory–inhibitory signal, scaling with accumulated evidence (cognitive load), to suppress that representation; the load-dependent inhibition of value coding is the substantive hypothesis. By contrast, the spiking substrate, the leaky integrate-and-fire neurons, the specific NEF/Nengo realization, and the particular basal-ganglia action-selection module are implementation choices. They confer biological plausibility and, crucially, let us read out population-level neural trajectories and spike rasters that can be confronted with electrophysiology and pupillometry – but the qualitative behavioral effect does not strictly require spikes. A rate-based neural network implementing the same load-dependent gain modulation would likely reproduce the cue-acquisition effect; spiking dynamics become necessary only when the goal is to relate the model to spike-level neural data and noise. A standard drift-diffusion model, as ordinarily formulated, cannot represent multiple interacting value populations or a neuromodulatory pathway acting on value coding; however, an extended sequential-sampling model with a time- or load-dependent collapsing bound (or input gain) could plausibly capture the behavioral reduction in sampling. What such reduced models would forgo is the mechanistic, neurally grounded interpretation—which population and which pathway implement the effect–and the ability to generate testable neural predictions. Our use of an SNN within the NEF is therefore best understood as a choice motivated by biological interpretability and linkage to neural data, rather than as a computational necessity for the behavioral result.

## Data Availability

The data presented in the study are deposited in the Open Science Framework (OSF) repository, https://osf.io/rnk8z, accession number rnk8z.
