# Supplementary material for: The role of inhibition in modeling decision making with spiking neural networks
Source: Front Neuroinform. 2026 Aug 12;20:1854811. doi: 10.3389/fninf.2026.1854811 (PMC13506930; doi:10.3389/fninf.2026.1854811)
Supplement: Supplementary file 1 [file Table_1.docx]

**Supplementary Table S1**

Per-participant comparison of No-Inhibition (NI) and Inhibition (I) model performance for the NC and C conditions, for all 24 participants. Loss values are reported as mean ± SD across three independent optimisation seeds. Bold indicates the model with lower mean loss for each participant and condition. Strategy preferences are majority votes across seeds using the discriminating-trial classification method (see main text, Methods).

| **ID** | **Cond.** | **Human Strategy** | **Loss NI (mean ± SD)** | **Loss I (mean ± SD)** | **Model NI** | **Model I** |
| --- | --- | --- | --- | --- | --- | --- |
| 2 | NC | TTB | **0.034 ± 0.005** | 0.052 ± 0.021 | WADD | TTB |
|  | C | WADD | 0.329 ± 0.066 | **0.301 ± 0.069** | TTB | TTB |
| 3 | NC | TTB | **0.055 ± 0.008** | 0.068 ± 0.047 | WADD | WADD |
|  | C | WADD | 0.354 ± 0.102 | **0.311 ± 0.055** | WADD | WADD |
| 4 | NC | TTB | **0.020 ± 0.012** | 0.042 ± 0.014 | WADD | WADD |
|  | C | TTB | 0.276 ± 0.067 | **0.228 ± 0.048** | WADD | WADD |
| 5 | NC | WADD | 0.259 ± 0.021 | **0.067 ± 0.019** | WADD | WADD |
|  | C | WADD | 1.438 ± 0.211 | **0.912 ± 0.065** | WADD | WADD |
| 6 | NC | TTB | **0.221 ± 0.046** | 0.453 ± 0.184 | WADD | WADD |
|  | C | WADD | 0.674 ± 0.063 | **0.255 ± 0.269** | WADD | TTB |
| 7 | NC | TTB | 0.206 ± 0.051 | **0.041 ± 0.021** | WADD | WADD |
|  | C | TTB | 1.161 ± 0.443 | **0.845 ± 0.152** | WADD | WADD |
| 8 | NC | TTB | 0.189 ± 0.189 | **0.023 ± 0.004** | WADD | WADD |
|  | C | TTB | **0.310 ± 0.095** | 0.397 ± 0.105 | WADD | WADD |
| 9 | NC | WADD | 0.000 ± 0.000 | **0.000 ± 0.000** | TTB | TTB |
|  | C | WADD | **1.484 ± 0.048** | 1.541 ± 0.051 | TTB | TTB |
| 10 | NC | TTB | 0.067 ± 0.013 | **0.035 ± 0.006** | WADD | WADD |
|  | C | TTB | **0.059 ± 0.008** | 0.065 ± 0.015 | TTB | TTB |
| 11 | NC | TTB | **0.027 ± 0.010** | 0.051 ± 0.021 | WADD | WADD |
|  | C | WADD | 1.063 ± 0.054 | **0.916 ± 0.145** | TTB | TTB |
| 12 | NC | WADD | **0.132 ± 0.080** | 0.153 ± 0.091 | WADD | WADD |
|  | C | WADD | 0.405 ± 0.110 | **0.283 ± 0.089** | WADD | WADD |
| 13 | NC | WADD | 0.022 ± 0.016 | **0.006 ± 0.006** | WADD | WADD |
|  | C | WADD | 0.494 ± 0.009 | **0.449 ± 0.076** | WADD | WADD |
| 14 | NC | WADD | 0.195 ± 0.145 | **0.045 ± 0.015** | WADD | WADD |
|  | C | WADD | 0.648 ± 0.305 | **0.267 ± 0.020** | WADD | WADD |
| 19 | NC | WADD | 0.211 ± 0.009 | **0.019 ± 0.013** | WADD | WADD |
|  | C | WADD | 0.348 ± 0.030 | **0.338 ± 0.092** | WADD | WADD |
| 20 | NC | TTB | **0.189 ± 0.035** | 0.410 ± 0.208 | WADD | WADD |
|  | C | TTB | 0.777 ± 0.019 | **0.607 ± 0.228** | TTB | TTB |
| 21 | NC | TTB | **0.039 ± 0.004** | 0.138 ± 0.162 | WADD | WADD |
|  | C | TTB | **0.598 ± 0.091** | 0.620 ± 0.088 | WADD | TTB |
| 22 | NC | WADD | 0.110 ± 0.047 | **0.038 ± 0.005** | WADD | WADD |
|  | C | WADD | **0.117 ± 0.076** | 0.184 ± 0.028 | TTB | WADD |
| 23 | NC | TTB | **0.035 ± 0.002** | 0.210 ± 0.293 | WADD | WADD |
|  | C | TTB | 0.819 ± 0.191 | **0.774 ± 0.220** | TTB | TTB |
| 24 | NC | TTB | **0.029 ± 0.015** | 0.082 ± 0.111 | WADD | WADD |
|  | C | TTB | 1.003 ± 0.144 | **0.819 ± 0.141** | TTB | TTB |
| 25 | NC | TTB | **0.001 ± 0.002** | 0.020 ± 0.018 | WADD | WADD |
|  | C | TTB | **1.071 ± 0.048** | 1.157 ± 0.052 | TTB | TTB |
| 26 | NC | TTB | **0.088 ± 0.005** | 0.496 ± 0.341 | WADD | WADD |
|  | C | TTB | **0.203 ± 0.037** | 0.258 ± 0.114 | TTB | WADD |
| 27 | NC | WADD | 0.204 ± 0.012 | **0.141 ± 0.037** | TTB | WADD |
|  | C | WADD | **0.385 ± 0.097** | 0.412 ± 0.133 | TTB | TTB |
| 30 | NC | WADD | 0.318 ± 0.061 | **0.255 ± 0.033** | WADD | WADD |
|  | C | TTB | 0.482 ± 0.190 | **0.162 ± 0.017** | TTB | WADD |
| 31 | NC | TTB | **0.109 ± 0.035** | 0.280 ± 0.270 | WADD | WADD |
|  | C | TTB | 1.546 ± 0.000 | **1.166 ± 0.623** | TTB | TTB |
